# Supplementary material for: Post-replicative pairing of sister ter regions in Escherichia coli involves multiple activities of MatP
Source: Nat Commun. 2020 Jul 30;11:3796. doi: 10.1038/s41467-020-17606-6 (PMC7394560; doi:10.1038/s41467-020-17606-6)
Supplement: Supplementary file 3 — Description of Additional Supplementary Files [file 41467_2020_17606_MOESM3_ESM.pdf]

Description of Additional Supplementary Files

File Name: Supplementary Data 1
Description: Supplementary Data 1 describes the number of foci detected for each category (1 or 2 foci/cell, FL or FH), depending on the strain used. The percentage this represents is indicated between brackets. Each category is further divided depending on the sub-cellular localisation of the focus: near mid-cell (within 1/6th cell length of mid-cell, M) or beyond 1/6th cell length, R), as explained in the main text.

File Name: Supplementary Data 2
Description: Supplementary Data 2 presents the value of the median for each parameter, depending on the strain and type of foci (1 or 2 foci/cell, FL or FH). Please refer to Figs 2d, 3b-c and Supplementary Figs 4 and 5 for a box plot representation of the parameters.

File Name: Supplementary Data 3
Description: Supplementary Data 3 presents the p-values of a two-sample Kolmogorov-Smirnov test for the mobility parameters of the foci for two strains: T4*matPΔ20* and T4*ΔzapB*. The test has been run either between the two strains, in which case the population of foci that has been compared is the one located at mid-cell (M, top 2 rows); or within the same strain, between foci located at mid-cell (M) or away from mid-cell (R) (bottom 2 rows). The results of other tests run are indicated in the main text.
