# Supplementary material for: Post-replicative pairing of sister ter regions in Escherichia coli involves multiple activities of MatP
Source: Nat Commun. 2020 Jul 30;11:3796. doi: 10.1038/s41467-020-17606-6 (PMC7394560; doi:10.1038/s41467-020-17606-6)
Supplement: Supplementary file 7 — Reporting Summary [file 41467_2020_17606_MOESM7_ESM.pdf]

nature research

Corresponding author(s): Estelle Crozat

Last updated by author(s): Jun 11, 2020

Reporting Summary

Nature Research wishes to improve the reproducibility of the work that we publish. This form provides structure for consistency and transparency in reporting. For further information on Nature Research policies, see Authors & Referees and the Editorial Policy Checklist .

Statistics

For all statistical analyses, confirm that the following items are present in the figure legend, table legend, main text, or Methods section.

n/a	Confirmed	
☐	☒	The exact sample size (n) for each experimental group/condition, given as a discrete number and unit of measurement
☐	☒	A statement on whether measurements were taken from distinct samples or whether the same sample was measured repeatedly
☒	☐	The statistical test(s) used AND whether they are one- or two-sided<br>*Only common tests should be described solely by name; describe more complex techniques in the Methods section.*
☒	☐	A description of all covariates tested
☒	☐	A description of any assumptions or corrections, such as tests of normality and adjustment for multiple comparisons
☐	☒	A full description of the statistical parameters including central tendency (e.g. means) or other basic estimates (e.g. regression coefficient) AND variation (e.g. standard deviation) or associated estimates of uncertainty (e.g. confidence intervals)
☒	☐	For null hypothesis testing, the test statistic (e.g. F, t, r) with confidence intervals, effect sizes, degrees of freedom and P value noted<br>*Give P values as exact values whenever suitable.*
☒	☐	For Bayesian analysis, information on the choice of priors and Markov chain Monte Carlo settings
☒	☐	For hierarchical and complex designs, identification of the appropriate level for tests and full reporting of outcomes
☒	☐	Estimates of effect sizes (e.g. Cohen's d, Pearson's r), indicating how they were calculated

Our web collection on statistics for biologists contains articles on many of the points above.

Software and code

Policy information about availability of computer code

Data collection	Microscopy data were collected using custom code to run the microscope, stage, and camera.
Data analysis	Data were analysed using custom Matlab R2018a codes that have been published already (Javer et al, 2013 and Amitai et al, 2017). They are available at https://github.com/ver228/bacteria-loci-tracker and at http://bionewmetrics.org/ ; "Nuclear Organization" section, respectively.

For manuscripts utilizing custom algorithms or software that are central to the research but not yet described in published literature, software must be made available to editors/reviewers. We strongly encourage code deposition in a community repository (e.g. GitHub). See the Nature Research guidelines for submitting code & software for further information.

Data

Policy information about availability of data

All manuscripts must include a data availability statement. This statement should provide the following information, where applicable:
- Accession codes, unique identifiers, or web links for publicly available datasets
- A list of figures that have associated raw data
- A description of any restrictions on data availability

The data that support the findings of this study are available from the corresponding author upon reasonable request.

Field-specific reporting

Please select the one below that is the best fit for your research. If you are not sure, read the appropriate sections before making your selection.

☒

Life sciences study design

All studies must disclose on these points even when the disclosure is negative.

Sample size	At least 3 biological replicates for each strain were run, leading n>3000 foci/strain. For each strain, the replicates were compared and checked if reproducible. Sample sizes exceeded standards in the field, which is usually a few hundreds of foci.
Data exclusions	Collected data were reproducible and therefore none was excluded.
Replication	All experiments were repeated at least 3 times and were reproducible.
Randomization	Our study is based on a computer analysis of foci movements and biochemical datas. Randomization is not relevant here.
Blinding	Our study is based on a computer analysis of foci movements and biochemical datas. Blinding is not relevant here.

Reporting for specific materials, systems and methods

We require information from authors about some types of materials, experimental systems and methods used in many studies. Here, indicate whether each material, system or method listed is relevant to your study. If you are not sure if a list item applies to your research, read the appropriate section before selecting a response.

Materials & experimental systems

n/a	Involved in the study
☒	☐ Antibodies
☒	☐ Eukaryotic cell lines
☒	☐ Palaeontology
☒	☐ Animals and other organisms
☒	☐ Human research participants
☒	☐ Clinical data

Methods

n/a	Involved in the study
☒	☐ ChIP-seq
☒	☐ Flow cytometry
☒	☐ MRI-based neuroimaging
