# Supplementary material for: Post-replicative pairing of sister ter regions in Escherichia coli involves multiple activities of MatP
Source: Nat Commun. 2020 Jul 30;11:3796. doi: 10.1038/s41467-020-17606-6 (PMC7394560; doi:10.1038/s41467-020-17606-6)
Supplement: Supplementary file 8 — Source Data [file 41467_2020_17606_MOESM8_ESM.zip › RawTPMData_README.docx]

**Raw TPM data used in Fig6 and Supp Fig8**

|  | **DNA** | **PROTEINS** | **DATES** | **NUMBER**  **For 5 min** | |
| --- | --- | --- | --- | --- | --- |
| Fig. 6 | *2 MatS* | MatP | 20140716 – Ch7-1  20140722 – Ch1-1  20140722 – Ch1-2 | 126  264  140 | Total= 530 |
|  | *1 MatS* | MatP | 20140829 – 1  20140829 – 2 | 146  185 | Total= 331 |
|  | - | MatP | 20140716 – Ch3-2  20140722 – Ch5-1 | 277  310 | Total= 587 |
|  | *2 MatS* | MatP-delta20 | 20140722 – Ch6-2 | 235 | Total= 235 |
| Fig. S8 | *2 MatS* | MatP + competitor DNA | 20140320-ch1b  20140320-ch1h  20140320-ch4b | 246  218  254 | Total= 718 |

**Content of Files ResultsParticleN.csv**

| X | Y | Xanc | Yanc | SymFact | Aeq | Aeqanc |
| --- | --- | --- | --- | --- | --- | --- |
| 75772,9 | 2620,83 | 75957,5 | 2854,43 | 1,11969 | 0 | 0 |

X: absolute centroid X-position of the tethered particle in nm

Y: absolute centroid Y-position of the tethered particle in nm

Xanc: absolute X-position of the DNA anchoring point in nm, calculated as the average of X on a 2s window

Yanc: absolute Y-position of the DNA anchoring point in nm, calculated as the average of Y on a 2s window

with N_fen_=51 images acquired at 25 Hz

SymFact : ratio of the maximum value to the minimum value of the eigenvalue of the covariance matrix of (X-Xanc, Y-Yanc) (details in Blumberg,S., Gajraj,A., Pennington,M.W. and Meiners,J.-C. (2005) Three-Dimensional Characterization of Tethered Microspheres by Total Internal Reflection Fluorescence Microscopy. Biophys J, 89, 1272–1281)

Aeq: square root of the mean square distance of the tethered particle to its anchoring point

Aeqanc: square root of the mean square distance of the anchoring point to the initial anchoring point

**Content of ParticulesSELECTtriNparticules.txt :** list of the selected tethered particles

Filename	Ptest1	SymFact	Pinvalid1	Pinvalid2

With

Ptest1: Aeq measured before injection of MatP should be contained in a gaussian centred on the mean value ((µ) ) of Aeq calculated for the ensemble of tracked particles and with a standard deviation (µ). Ptest1=1 if these initial Aeq belongs to [µ -2.5 σ ; µ +2.5 σ ]

Pinvalid1: ratio of images where Aeq<1 , is required to be equal to 0

Pinvalid2: ratio of images where Aeq<100, is required to be inferior to 0.1
